# Influence of the Synthesis Scheme of Nanocrystalline Cerium Oxide and Its Concentration on the Biological Activity of Cells Providing Wound Regeneration

**DOI:** 10.3390/ijms241914501

**Published:** 2023-09-24

**Authors:** Ekaterina V. Silina, Victor A. Stupin, Natalia E. Manturova, Olga S. Ivanova, Anton L. Popov, Elena A. Mysina, Elena B. Artyushkova, Alexey A. Kryukov, Svetlana A. Dodonova, Maria P. Kruglova, Alexey A. Tinkov, Anatoly V. Skalny, Vladimir K. Ivanov

**Affiliations:** 1 Institute of Biodesign and Modeling of Complex Systems, Center of Bioelementology and Human Ecology, I.M. Sechenov First Moscow State Medical University (Sechenov University), 119991 Moscow, Russia; marykruglova@live.ru (M.P.K.); tinkov.a.a@gmail.com (A.A.T.); skalny3@gmail.com (A.V.S.); 2 Department of Hospital Surgery, Pirogov Russian National Research Medical University, 117997 Moscow, Russia; stvictor@bk.ru; 3 Department of Plastic and Reconstructive Surgery, Cosmetology and Cell Technologies, Pirogov Russian National Research Medical University, 117997 Moscow, Russia; manturovanatali@yandex.ru; 4 Frumkin Institute of Physical Chemistry and Electrochemistry, Russian Academy of Sciences, 119071 Moscow, Russia; runetta05@mail.ru; 5 Institute of Theoretical and Experimental Biophysics, Russian Academy of Sciences, 142290 Pushchino, Russia; antonpopovleonid@gmail.com (A.L.P.); mironova_e27@rambler.ru (E.A.M.); 6 Research Institute of Experimental Medicine, Kursk State Medical University, 305041 Kursk, Russia; eartyushkova@mail.ru (E.B.A.); krukovaa@kursksmu.net (A.A.K.); dodonovasa@kursksmu.net (S.A.D.); 7 Laboratory of Ecobiomonitoring and Quality Control, Yaroslavl State University, 150003 Yaroslavl, Russia; 8 Kurnakov Institute of General and Inorganic Chemistry, Russian Academy of Sciences, 119991 Moscow, Russia; van@igic.ras.ru

**Keywords:** cerium oxide nanoparticles, nanocerium, nanoceria, fibroblasts, mesenchymal stem cells, keratinocytes, metabolism, proliferation, cytotoxicity

## Abstract

In the ongoing search for practical uses of rare-earth metal nanoparticles, cerium dioxide nanoparticles (nanoceria) have received special attention. The purpose of this research was to study the biomedical effects of nanocrystalline forms of cerium oxide obtained by different synthesis schemes and to evaluate the effect of different concentrations of nanoceria (from 10^−2^ to 10^−6^ M) on cells involved in the regeneration of skin cell structures such as fibroblasts, mesenchymal stem cells, and keratinocytes. Two different methods of nanoceria preparation were investigated: (1) CeO-NPs-1 by precipitation from aqueous solutions of cerium (III) nitrate hexahydrate and citric acid and (2) CeO-NPs-2 by hydrolysis of ammonium hexanitratocerate (IV) under conditions of thermal autoclaving. According to the X-ray diffraction, transmission electron microscopy, and dynamic light scattering data, CeO_2_-1 consists of individual particles of cerium dioxide (3–5 nm) and their aggregates with diameters of 60–130 nm. CeO_2_-2 comprises small aggregates of 8–20 nm in diameter, which consist of particles of 2–3 nm in size. Cell cultures of human fibroblasts, human mesenchymal stem cells, and human keratinocytes were cocultured with different concentrations of nanoceria sols (10^−2^, 10^−3^, 10^−4^, 10^−5^, and 10^−6^ mol/L). The metabolic activity of all cell types was investigated by MTT test after 48 and 72 h, whereas proliferative activity and cytotoxicity were determined by quantitative cell culture counting and live/dead test. A dependence of biological effects on the method of nanoceria preparation and concentration was revealed. Data were obtained with respect to the optimal concentration of sol to achieve the highest metabolic effect in the used cell cultures. Hypotheses about the mechanisms of the obtained effects and the structure of a fundamentally new medical device for accelerated healing of skin wounds were formulated. The method of nanoceria synthesis and concentration fundamentally and significantly change the biological activity of cell cultures of different types—from suppression to pronounced stimulation. The best biological activity of cell cultures was determined through cocultivation with sols of citrate nanoceria (CeO-NPs-1) at a concentration of 10^−3^–10^−4^ M.

## 1. Introduction

Recent decades have been marked by the rapid development of new technologies in various fields. Often, these new discoveries overturn our previous ideas about the laws of the world around us. One such trend has been the creation of nanoparticles, many of the properties of which cannot be explained by older trivial notions of chemical or physical interactions [1,2,3]. This has resulted in prerequisites for the creation of materials with new properties and the search for applications for these newly discovered properties to solve complex technical or social problems. One such a problem is the rapid regeneration of skin via different mechanisms following damage in acute and especially chronic wounds. The number of elderly patients with diabetes mellitus and chronic venous and arterial insufficiency with the formation of trophic ulcers exhibits a constant progressive trend [4,5]. Relentlessly recurrent and requiring constant and expensive treatment, such diseases are accompanied by progressively increasing costs to government and family budgets [6,7]. This silent epidemic takes more lives and resources annually than the recent SARS-CoV-19 pandemic [8,9,10,11,12]. The situation with these diseases challenges us to find alternative solutions for cost-effective and biologically determined treatment as soon as possible. According to research on the wound care market, thanks to newly developed solutions, patients with chronic wounds are already receiving care that is safe, effective, and promotes rapid recovery [13,14,15]. In this context, the growing field of nanobiotechnology can provide an alternative platform for the development of new therapeutic agents for the treatment of wound infections and early regeneration of acute and chronic wounds, taking into account possible risks [16].

The purpose of the presented work is to demonstrate the first steps in the development of a new agent based on nanomaterials, providing methods for faster healing and demonstrating differentiated capabilities as a new type of antimicrobial agent [17,18,19,20,21,22,23,24,25].

Metal nanoparticles, especially those of variable-valence metals, have been considered for clinical applications in this field due to their high levels of activity in biologically relevant redox processes and their ability to regulate the concentration of reactive oxygen species in living systems. Metal oxide nanoparticles are advantageous due to their high surface area-to-volume ratio, as well as their stability and safety.

At present, the application of nanotechnology for actual use in health care and basic research on the interaction of nanomaterials with cells and their biological effects in macro-organisms is just beginning [1,26,27]. Of the many materials that have been widely reported in the modern literature with highly probable pharmacological potential, the most studied is nanoscale cerium oxide, which realizes several biologically beneficial effects at once [27,28,29]. Therefore, cerium oxide nanoparticles (CeO-NPs) are presented as ideal candidates for wound application, as well as for integration into dressings and dressing material [30,31,32,33,34].

A number of studies have established proliferative [32,35,36,37], antimicrobial [33,38,39,40], antioxidant [41,42], and antitumor effects of nanoceria [43,44]. However, the presence and intensity of these effects vary considerably across reports from different investigators. These effects depend on many objective factors, such as the pH of the medium, particle size and concentration, exposure temperature, stoichiometry, and ligand environment, and perhaps fundamental to the principal differences in the obtained effects is the method of nanoparticle synthesis [27,38,45,46,47]. 

One of the problems associated with creating drugs based on nanoparticles is that in the multitude of described methods of nanoceria synthesis, different authors often obtain effects that are difficult to compare. Considering that pure cerium dioxide is used as a guaranteed drug in all described cases, it can be assumed that the crucial factor is not the chemical formula but the physical and chemical characteristics the obtained nanoparticles. Therefore, the development of new nanodrugs should always start with an evaluation of the methodology of CeO-NPs synthesis, the physicochemical properties and biological effects of each particular compound, and, most importantly, the repeatability of the results in several independent research laboratories with different profiles.

The purpose of our work is the simultaneous study of physicochemical characteristics and biomedical effects of nanocrystalline cerium oxide obtained by two different methods (1—precipitation from aqueous solutions of cerium (III) nitrate hexahydrate and citric acid and 2—by hydrolysis of ammonium hexanitratocerate (IV) under conditions of thermal autoclaving), with evaluation of the effects of different concentrations of CeO-NPs sols (from 10^−2^ to 10^−6^ M) on metabolic and proliferative activity of different tissue layers involved in wound repair (on cell cultures of human fibroblasts, mesenchymal stem cells, and human keratinocytes) for the subsequent creation of a medical device stimulating regeneration.

## 2. Results

### 2.1. Evaluation of the Effect of Different Synthesis Methods and Different Concentrations of Nanocrystalline Cerium Oxide on Cytotoxicity and Metabolic and Proliferative Activity of Human Fibroblast Cell Culture

The MTT tests revealed that citrate nanocrystalline cerium oxide CeO-NPs-1 significantly stimulated fibroblast metabolism in a large range of concentrations, namely at doses of 10^−3^, 10^−4^, and 10^−5^ M by an average of 34%, 26%, and 14%, respectively, compared to the control (*p* < 0.01). The highest concentration (10^−2^ M) and the lowest concentration (10^−6^ M) had no significant effect compared to the control group.

In addition, statistically significant differences between subgroups with different concentrations of nanocerium CeO-NPs-1 were determined. Thus, the best stimulation of fibroblasts metabolism (the highest level according to MTT test) was in the CeO-NPs-1 sample with a concentration of 10^−3^ M, for which the index of optical cells was statistically significantly higher than at all other concentrations of CeO_2_ (*p* < 0.01). The second most effective among all concentrations of CeO-NPs-1 was the 10^−4^ M dilution (*p* < 0.01); the OD level was statistically significantly higher than at 10^−2^, 10^−5^, 10^−6^ M dilutions and in the control. Although the sample with a 10^−5^ M concentration exhibited significant stimulation relative to the control, this sample did not statistically differ from the cerium oxide sample at the maximum dilution of 10^−6^ M.

The results of the MTT test when fibroblasts were cocultured with nanoceria obtained by hydrolysis method (CeO-NPs-2) showed differing directionality of effect. CeO-NPs-2 at a concentration of 10^−2^ M had a suppressive effect on the proliferative activity of the studied cell line (an average of −21% relative to the control (*p* < 0.05)). In contrast, CeO_2_ at concentrations of 10^−4^ M and 10^−6^ M stimulated the proliferative activity of the cells, with the percentage of cells with adequate respiratory activity increased by an average of 20% and 31%, respectively. However, intermediate CeO-NPs-2 concentrations of 10^−3^ M and 10^−5^ M did not show convincing stimulation and did not statistically differ from the control. Significant differences from all other subgroups were established in the 10^−2^ M sample, with an OD value that was statistically significantly lower than that of all other subgroups for all syntheses and concentrations of nanocerium, demonstrating cytotoxicity. The best result was shown by the 10^−6^ M sample, which was significantly higher than the others in this group but not statistically different from the 10^−4^ M concentration.

**Figure 1:**
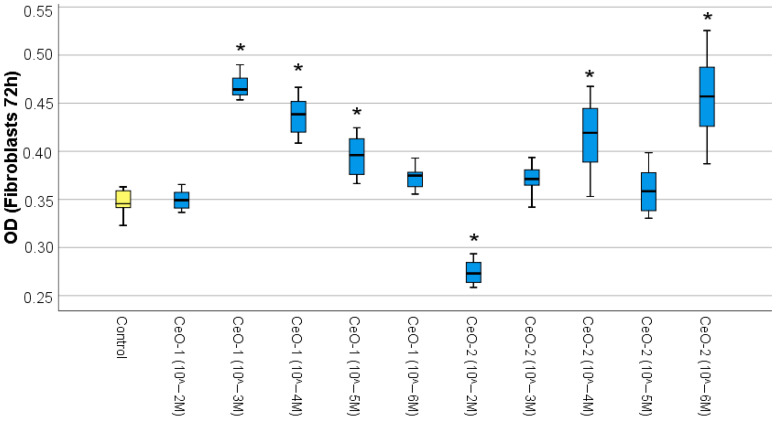
Effect of different concentrations of cerium oxides on the metabolic activity of fibroblasts in the MTT test (difference relative to the control at * *p* < 0.001; ANOVA).

ANOVA revealed a significant difference in the studied samples (OD: F = 77.26; *p* < 0.0001) (Figure 1; Table 1).

According to Duncan’s test data, 7 homogeneous subsets were formed from 11 samples (control, five dilutions of CeO-NPs-1, and five dilutions of CeO-NPs-2). The best stimulation of fibroblast metabolism (six to seven subsets) according to the MTT test data was determined in the CeO-NPs-1 samples at concentrations of 10^−3^ M and 10^−4^ M, as well as in the CeO-NPs-2 sample at a concentration of 10^−6^ M.

A study of proliferative activity of fibroblasts according to the results of quantitative cell counting showed the absence of a stimulating effect of both samples of nanocerium sols. Statistical evaluation of the obtained results using one-factor analysis of variance (ANOVA) showed the presence of statistically significant changes in the experimental groups (F = 7.79; *p* < 0.001). The number of fibroblasts in wells after 72 h when cocultured with CeO-NPs-1 at concentrations of 10^−2^, 10^−3^, 10^−4^, and 10^−6^ M was found to be significantly lower than in control wells by an average of 61%, 42%, 39%, and 45%, respectively (*p* < 0.01). When cocultured with CeO-NPs-2 at concentrations of 10^−2^ M and 10^−5^ M, the number of fibroblasts was significantly lower than the control by an average of 54% and 36%, respectively (*p* < 0.01). 

**Figure 2:**
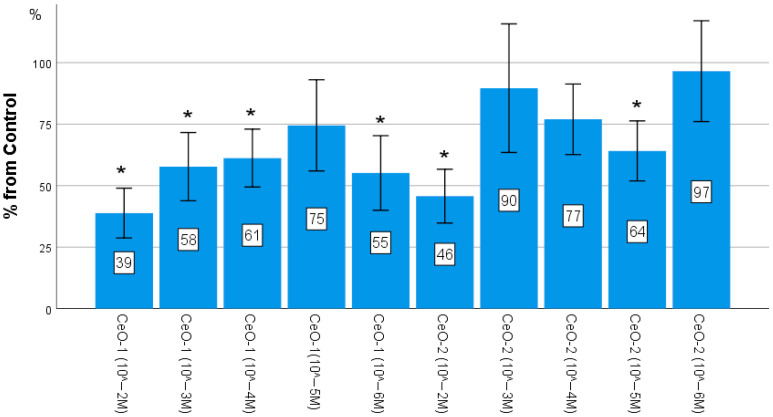
Effect of different concentrations of cerium oxide on the proliferative activity of fibroblasts determined by direct cell counting using an automated cell counter. The mean percentage value relative to the control is presented. Error columns: 95I for the mean (ANOVA (OD: F = 7.789; *p* < 0.001; difference from control at * *p* < 0.01 according to Dunnett’s test)).

Thus, the worst result was obtained in both samples at the 10^−2^ M concentration, while the best result (statistically comparable to the norm) was recorded in the CeO-NPs-1 sample at a 10^−5^ M concentration and in the CeO-NPs-2 sample at a concentration of 10^−6^ M (Figure 2, Table 2).

Despite the fact that metabolic activity of fibroblasts in the process of regeneration with the synthesis of interstitial intercellular substances is more important than their proliferation, the risk of cytotoxicity development at high concentrations of nanocerium CeO-NPs-1 and CeO-NPs-2 sols was determined. Thus, after 72 h when cocultured with nanocerium sols at concentrations of 10^−2^ M CeO-NPs-1 and 10^−2^ M CeO-NPs-2, the average number of fibroblasts was 53 thousand and 62 thousand cells, respectively, while the control had 136 thousand fibroblasts (2.6 times and 2.2 times higher, respectively; *p* < 0.01).

Evaluating the ratio of live to dead cells using trypan blue revealed no statistically significant differences between the experimental groups. Dead cells were absent in most cases; they were found in single wells in minimal amounts (1–5%), occurring unreliably more often in association with high concentrations of CeO-NPs-2. This evidence suggests a high level of biosafety of cerium dioxide nanoparticles at all concentrations we studied.

Since high concentrations of both samples of cerium oxide (10^−2^ M) can inhibit both metabolic and proliferative activity of fibroblasts, these concentrations are inappropriate to use for the creation of regenerative mediastinum. Therefore, further experiments on MSCs and keratinocytes were performed at sol concentrations of 10^−3^–10^−6^ M.

### 2.2. Assessment of the Effect of Different Synthesis Methods and Concentrations of Nanocrystalline Cerium Oxide on Cytotoxicity and Metabolic and Proliferative Activity of Cell Culture of Human Mesenchymal Stem Cells

According to MTT test data on human MSC cultures, after 48 h, a significant stimulation of metabolism of both sol samples at concentrations 10^−4^ M–10^−5^ M was detected. Specifically, the OD level of MSC culture following the addition of CeO-NPs-1 at a concentration of 10^−4^ M was, on average, 1.12 times higher than in the control (*p* < 0.05) and 18 times higher at a concentration of 10^−5^ M (*p* < 0.05). Following the addition of CeO-NPs-2 at concentrations of 10^−4^ M and 10^−5^ M, the OD level was 1.13 times higher than in the control (*p* < 0.01).

After 72 h, MSCs were found to be inhibited by CeO-NPs-2 at a concentration of 10^−3^ M (average OD was 1.19-fold higher in the control, *p* < 0.05), and significant stimulation was determined only at a single concentration of 10^−4^ M CeO-NPs-2 (average of 1.12-fold, *p* < 0.05). The rest of the samples did not differ from the control. After 72 h of MSC cocultivation with CeO-NPs-1 sols, a statistically significant stimulating effect was found in samples at a concentration of 10^−5^ M (1.12 times, *p* < 0.05), and a tendency toward stimulation at a concentration of 10^−4^ M was determined.

Subsets were determined according to Duncan’s test data, and the best (after 48 h and after 72 h) were both types of nanocerium samples at concentrations of 10^−4^–10^−5^ M. Thus, these are the preferred concentrations when creating a drug to stimulate the metabolic activity of MSCs.

**Figure 3:**
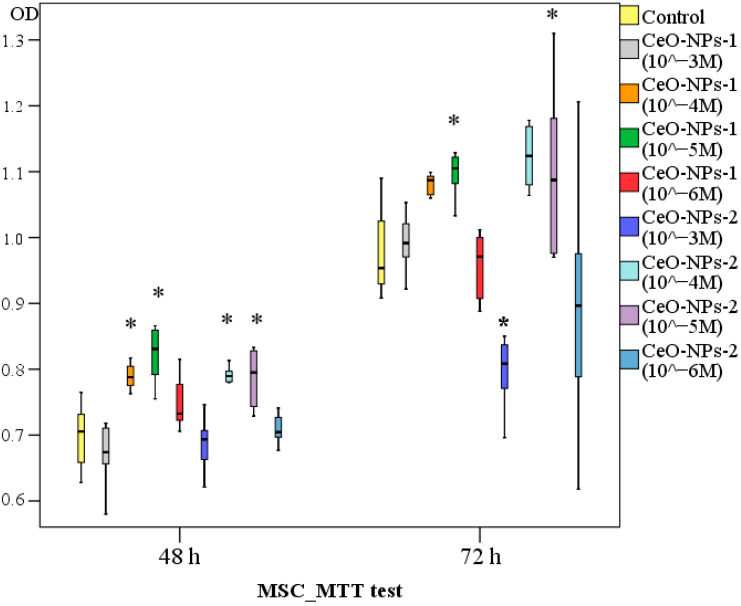
Effect of different concentrations of nanocerium samples on metabolic activity of human mesenchymal stem cells after 48 and 72 h of cocultivation and MTT test (difference relative to the control at * *p* < 0.01; ANOVA).

ANOVA revealed a significant difference between the studied samples after 48 h (F = 16.63; *p* < 0.0001) and 72 h (F = 10.71; *p* < 0.0001) (Figure 3, Table 3). 

With regard to safety, the decrease in the metabolic activity of MSCs after 72 h when cocultured with CeO-NPs-2 samples at a concentration of 10^−3^ M, as well as half of the test results at concentrations of 10^−6^ M, draws attention.

**Figure 4:**
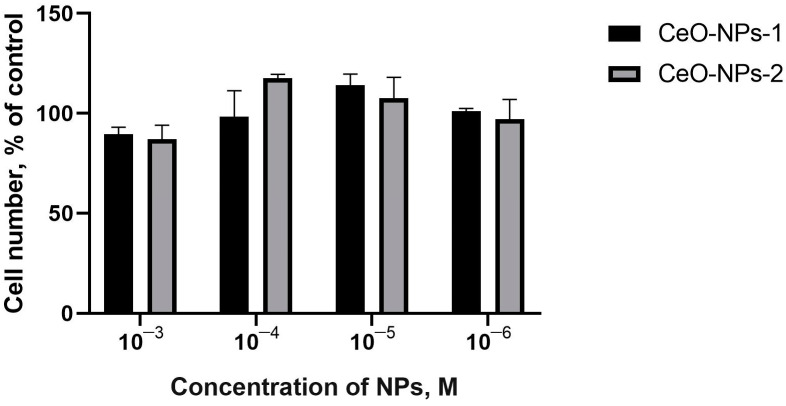
Human MSC cell culture counting data using cell counter 48 h after incubation with cerium oxide nanoparticles (CeO-NPs-1 and CeO-NPs-2).

**Figure 5:**
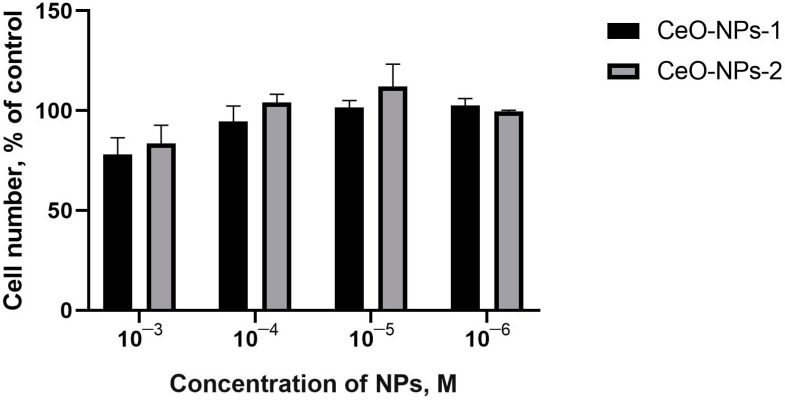
Human MSC culture counting data determined using a cell counter 72 h after incubation with cerium oxide nanoparticles (CeO-NPs-1 and CeO-NPs-2).

The results of quantitative counting of human MSCs using a counter showed that after 48 h, an increasing tendency with respect to the number of cells was detected only at a 10^−4^ M and 10^−5^ M concentration of both nanoceria (108–118%, *p* > 0.05) (Figure 4). After 72 h, an insignificant cell growth trend was determined only when coculturing with CeO-NPs-2 at a concentration of 10^−5^ M (112% on average, *p* > 0.05; 78–104% at other concentrations (Figure 5).

**Figure 6:**
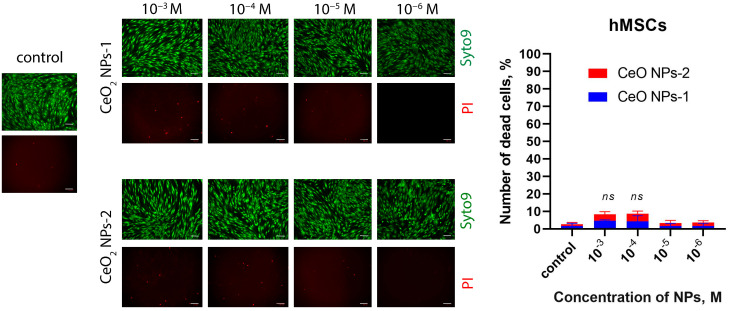
Staining of human MSC cultures with SYTO9 and propidium iodide fluorescent dyes after cultivation with nanoparticles for 72 h. Quantitative analysis of the number of dead cells in relation to the total number of cells. Scale bar—100 µm. ns—not significant via ANOVA test.

The percentage of dead cells in the MSC culture did not exceed 5%, with the highest biocompatibility in relation to MSCs (no more than 1% dead cells after 48 h and no detection of dead cells after 72 h) associated with CeO-NPs-1 sol samples at concentrations of 10^−4^ M and 10^−6^ M and CeO-NPs-2 at concentrations of 10^−4^ M and 10^−5^ M. The detection of dead MSCs in all tests were only detected in the CeO-NPs-2 sample at a concentration of 10^−3^ M after 72 h of cocultivation (Figure 6).

### 2.3. Evaluation of the Effect of Different Synthesis Methods and Different Concentrations of Nanocrystalline Cerium Oxide on Cytotoxicity and Metabolic and Proliferative Activity of Human Keratinocyte Cell Culture

According to MTT test data after 48 h of cocultivation with human keratinocyte culture, a significant stimulating effect of the CeO-NPs-1 sample at a concentration of 10^−3^ M was detected; the optical density of these samples was, on average, 2.17 times higher (OD Me = 0.787; IQR 0.66/0.87) than that of the control (*p* < 0.01) and significantly higher compared to all other subgroups. Other samples at all other concentrations were not statistically different from the control, varying in OD between 0.360 and 0.384, on average.

After 72 h of keratinocyte culture cocultivation with sols of nanocrystalline cerium oxide, a significant stimulating effect of CeO-NPs-1 samples at concentrations of 10^−3^ M and 10^−4^ M was revealed (OD averaged 1.44 and 1.12, respectively, which was 3.24-fold and 2.51-fold higher than in the control, respectively; *p* < 0.01). At the other concentrations (10^−5^–10^−6^ M) of CeO_2_-1 sols, a stimulation tendency was registered, with an average OD value of coculturing keratinocytes that was, on average, 1.4–1.6 times higher (41–62%) than that of the control (*p* > 0.05).

**Figure 7:**
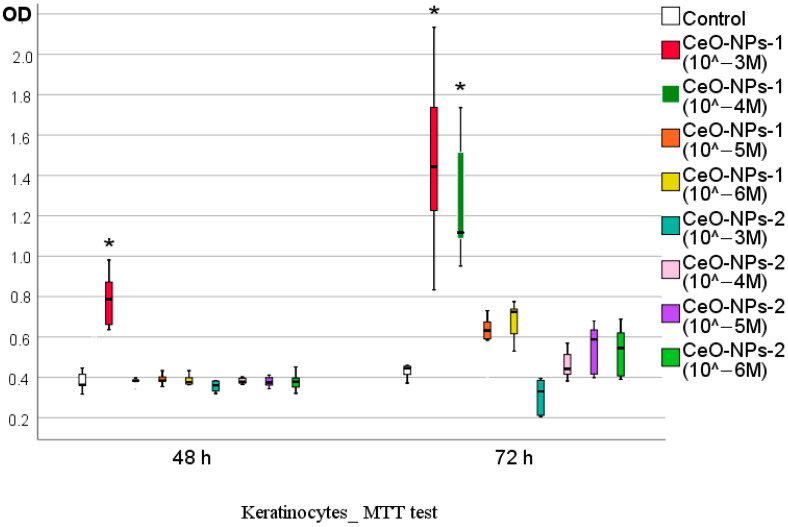
Effect of different concentrations of cerium oxides on metabolic activity of human keratinocytes after 48 and 72 h of cocultivation according to MTT test (difference from control at * *p* < 0.01; Kruskal–Wallis test).

MTT test data for samples of CeO-NPs-2 sols after 72 h (as well as after 48 h) showed no stimulating effect, with a decrease the OD index observed when keratinocytes were co-cultured with CeO-NPs-2 at a concentration of 10^−3^ M (OD in the control was, on average, 1.35 times higher, *p* > 0.05) (Figure 7, Table 4).

Comparative analysis of the results of quantitative keratinocyte counting did not demonstrate statistically significant differences from the control or between groups. After 48 h, the number of keratinocytes showed an increasing trend only at concentrations of 10^−4^ M CeO-NPs-2 (110% of control, *p* > 0.05); the other samples averaged 79–99% of the control value.

**Figure 8:**
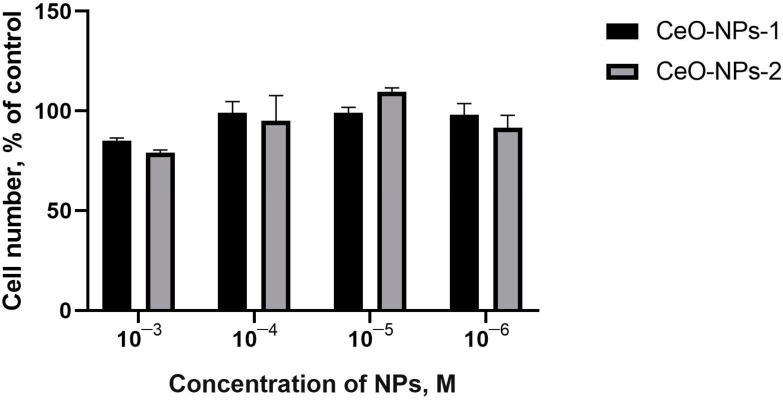
Human keratinocyte cell culture counting data collected using a cell counter 48 h after incubation with nanoparticles.

**Figure 9:**
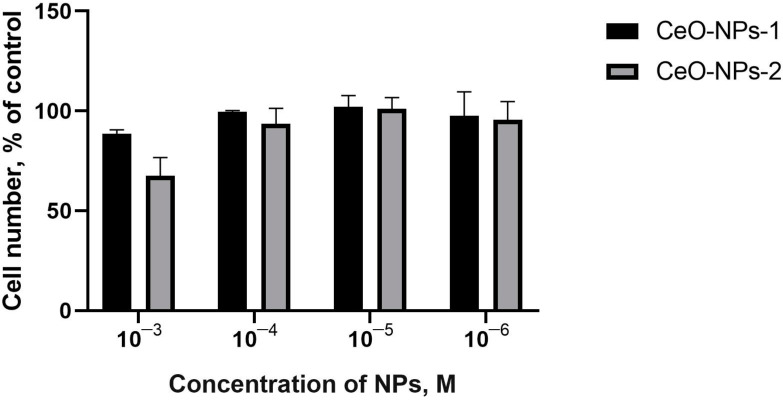
Human keratinocyte cell culture counting data collected using a cell counter 72 h after incubation with nanoceria.

**Figure 10:**
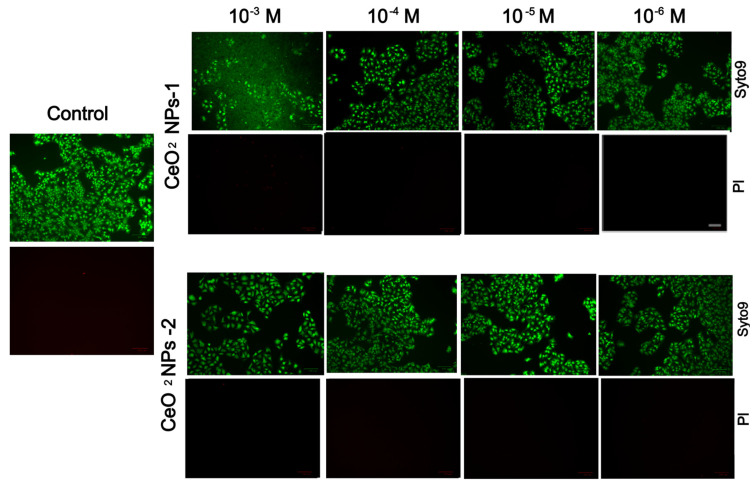
Staining of keratinocytes with SYTO9 and propidium iodide fluorescent dyes after cultivation with nanoparticles for 72 h. Quantitative analysis of the number of dead cells in relation to the total number of cells. Scale bar—100 µm.

After 72 h, the number of cells was either lower than or equal to that in the control (68–102%), and a trend towards a 32% reduction in the number of keratinocytes was determined when cocultured with CeO-NPs-2 at a concentration of 10^−3^ M (Figure 8, Figure 9 and Figure 10).

The percentage of dead cells in human keratinocyte culture did not exceed 5%, with the highest biocompatibility in relation to human keratinocytes (no more than 1% of dead cells after 48 h and no detection of dead cells after 72 h) associated with the CeO-NPs-1 sol samples at concentrations of 10^−4^ M and 10^−6^ M and CeO-NPs-2 samples at concentrations of 10^−4^ M and 10^−5^ M. In all tests, dead cells were only detected at high concentrations of CeO-NPs-2 (10^−3^ M) after 72 h (1–2% in all tests).

## 3. Discussion

The ongoing discussions in the biological and medical literature about the potential of cerium compounds to accelerate wound regeneration are supported by multidirectional research results [27,48]. This is explained largely by an incomplete understanding of the mechanisms of action of nanoparticles, the patterns of their interaction with the environment, and the construction of hypotheses that do not yet have rigorous scientific substantiation [44,49,50]. If we consider that physicochemical properties of nanoceria, such as particle size, agglomeration state in liquid, surface charge, etc., play an important role in the final interaction of nanoparticles with target cells, it becomes clear how difficult it is to compare the results obtained from different studies.

Recently, nanotechnology-based treatments have been trending with respect to the creation of new drugs and dressings. Some studies have shown promising results [51,52,53,54,55]. However, there are many problems associated with different technical solutions to obtain nanoceria. Differences between methods of nanoparticle production, as well as methods to ensure guaranteed particle size and surface chemistry, the long-term stability of sols, and the lack of control groups for objective comparison between studies lead to problems in choosing the optimal technical solution for the creation of a new type of medical device.

Many researchers have noted the dependence of the effects nanoceria on particle size [56,57,58], possibly due to their total area or some other variables that increase or decrease biocompatibility.

More than ten chemical and physical methods for the synthesis of cerium nanoparticles have been described in the literature. As a result, after washing and filtration, a chemically identical substance can be obtained. However, synthesis methods affect the physical characteristics of nanoceria, leading to biological effects of varying strength. 

We intend to solve the problem of choosing a specific method for the synthesis of CeO-NPs, which would allow us to obtain stable nanoparticles with maximum biologically useful properties for the regeneration of skin wounds. This work begins a cycle of studies on the comparative efficacy of each element of nanocerium-based compositions obtained by different methods for subsequent drug development for veterinary and clinical medicine. 

This manuscript presents the first comparative study involving unified biological experiments on the effect of solid cerium sols synthesized by two different chemical methods but with similar chemical composition (citrate-stabilized solid cerium solutions) and crystal size (~3 nm) on human fibroblasts, human mesenchymal stem cells, and human keratinocytes (cell cultures typical of skin tissue). Both synthetic protocols involve an aqueous chemical route and low reaction temperatures not exceeding 100 °C. Although different reagents (cerium (III) nitrate and cerium ammonium nitrate) are used to synthesize these cerium sols, both protocols yield nanocrystalline tetravalent cerium oxide. In addition, it is important to note that both cerium sols synthesized in our work have excellent stability, with no visible changes in their appearance observed for 2–3 months. According to our earlier data, both synthetic technologies allow us to obtain sols that remain stable for 1 year. The stability of sols was checked by dynamic light scattering, which allows us to obtain information on the particle size distribution and the degree of aggregation of nanoparticles. These are the main reasons for the choice of such methods for the synthesis of CeO-NPs.

When creating a product for medical use, the safety of cerium oxide nanoparticles is a fundamentally important aspect. That is why we studied cytotoxicity on different cells involved in wound healing. It is promising that we did not detect mass cell death in any of the performed studies. According to the analysis of the number of dead cells, we can conclude that the synthesized samples of cerium oxide have high biocompatibility, as they do not show a reliable increase in the proportion of dead cells in cultures of fibroblasts, MSCs, or human keratinocytes (the percentage of dead cells does not exceed 5%). CeO-NPs-1 sol samples at concentrations of 10^−4^ M and 10^−6^ M and CeO-NPs-2 sol samples at concentrations of 10^−4^ M and 10^−5^ M had the highest biocompatibility (no more than 1% dead cells after 48 h and no dead cells detected after 72 h in all tests). The presence of dead cells in all tests (although in a low percentage of cases, up to 5%) was observed only at high concentrations of CeO-NPs-2 (10^−2^ and 10^−3^ M) after 72 h.

At high concentrations (10^−2^ M) for both nanoceria samples, either suppression of proliferative and metabolic activity or comparability with the norm was registered (no stimulation was registered in any type of cell studied herein), and such concentrations can be considered cytotoxic. The risk of cytotoxicity was also established in CeO-NPs-2 samples at a concentration of 10^−3^ M. The mechanism of cytotoxicity is still not fully understood, but it may be associated with oxidative stress induced by many nanomaterials.

An important aspect of the series of cellular experiments was stimulation of metabolic activity, which was detected on MSCs, fibroblasts, and keratinocytes—that is, on all types of skin cells and, therefore, skin wounds.

The data obtained in this study indicate a pronounced stimulating effect of nanocrystalline cerium oxide obtained by the citrate method CeO-NPs-1 on the metabolic activity and viability of human fibroblasts (BJTERT cell line) in a wide range of concentrations from 10^−3^ to 10^−5^ M (average of 14–34%), with the best results achieved at concentrations of 10^−3^–10^−4^ M (activation increased by an average of 26–34%). Nanoceria at these concentrations significantly enhance metabolic processes in cell cultures of human fibroblasts and therefore promote the rapid creation of a sufficient amount of intercellular matrix for the transition of the wound into the regeneration phase.

Analysis of the results of in vitro studies of CeO-NPs-2 sols does support the selection of the best range of concentrations for future medical devices, since the revealed effect is nonlinear. However, given that high concentrations (10^−2^ M) demonstrated cytotoxicity and the best effect was observed at a concentration of 10^−6^ M, the most diluted colloidal solution of CeO-NPs-2 (10^−6^ M) may be preferred.

Human MSC culture after 48 h of cocultivation with nanoceria sols confirmed the stimulating activity in both samples at concentrations of 10^−4^–10^−5^ M; after 72 h, stimulation was observed only for CeO-NPs-2 at a concentration of 10^−4^ M and CeO-NPs-1 at a concentration 10^−5^ M, with a tendency toward better efficiency for both samples at concentrations of 10^−4^–10^−5^ M. However, the presence of 10^−3^ M CeO-NPs-2 after 72 h of co-cultivation depressed the metabolic activity of human MSCs; therefore, this concentration can be considered cytotoxic.

A significant stimulating effect of the CeO-NPs-1 samples at concentrations of 10^−3^–10^−4^ M (especially 10^−3^ M) in relation to human keratinocytes cell culture was also detected. Lower concentrations of this sample (10^−5^–10^−6^ M) also resulted in a significant increase in metabolic activity of human keratinocytes but only after 72 h of cocultivation with nanocerium sols. CeO-NPs-2 sols did not stimulate keratinocytes, and the CeO-NPs-2 sample at a concentration of 10^−3^ M showed a depressive (suppression) effect, which may also be a sign of cytotoxicity.

Thus, the general analysis of all results of MTT tests supports the creation of CeO-NPs-1 medical devices with concentrations in the range of 10^−3^–10^−5^ M (for stimulation of MSC metabolism an average of 1.1–1.2 times higher than with concentrations of 10^−4^–10^−5^ M; for stimulation of fibroblast metabolism an average of 1.2–1.3 times higher than with concentrations of 10^−3^–10^−4^ M; for stimulation of keratinocytes metabolism, an average of 2.2–3.2 times higher than with concentrations of 10^−3^–10^−4^ M). Based on all data, the most preferable concentration for stimulation of all layers of cell regeneration is 10^−3^–10^−4^ M (closer to 10^−4^ M, taking into account maximum biocompatibility and nontoxicity).

The proliferation rate of cells Involved in regeneration did not change under the influence of any of the studied variants of nanocerium sols. Therefore, we suppose that for acceleration of wound healing, which is impossible to achieve without increasing the number of cells included in the physiological structure of the skin, it is necessary to add components accelerating the processes of cell division to the medicinal forms. At the same time, a tendency to decrease their proliferation was determined on fibroblasts, which may be associated with the expenditure of cell energy on the stimulation of metabolism and synthesis of collagen and elastin, as shown in the experiment, but not on cell reproduction.

It should be noted that fibroblasts, unlike mesenchymal stem cells, have a finite number of divisions (Hayflick number); for the fibroblasts we used, this number was about 30–40 divisions. Since the main function of fibroblasts is the synthesis of collagen and other components of the intercellular matrix, the proliferation index, i.e., the increasing number and density of cells, is not a determinant problem associated with wound healing. It can be assumed that a high level of metabolic activity of even a small number of these resident cells in the wound triggers regeneration processes. The absence of cell death, along with a high level of metabolic activity, may indicate the effectiveness of cerium dioxide sols on cell cultures of fibroblasts and, potentially, for wound healing. The question remains open about a possible decrease in energy reserves for collagen synthesis in fibroblasts under the action of high concentrations of nanoceria, which is consistent with an increase in the number of fibroblasts not energetically depleted in the control group. However, this hypothesis can only be tested in further studies.

## 4. Materials and Methods

The objects of our biomedical studies were aqueous sols of nanocrystalline cerium oxide obtained by two different methods at five concentrations: 10^−2^, 10^−3^, 10^−4^, 10^−5^, and 10^−6^ mol/L.

Both types of nanocerium were obtained using previously published solution techniques. The first type of nanocerium (thermohydrolyzed CeO-NPs-1) was prepared from aqueous solutions of cerium (III) nitrate hexahydrate and citric acid [59]. The second type of nanocerium (CeO-NPs-2, citrate) was obtained by hydrolysis of ammonium hexanitratocerate (IV) under conditions of thermal autoclaving and additionally stabilized by ammonium citrate [60,61].

### 4.1. Research Design

This study is interdisciplinary and was conducted in different institutions, ensuring blinding to enhance its relevance.

In the first stage, samples of nanocerium sols were synthesized in sufficient quantity for all experiments and characterized.

In the second step, in vitro studies were carried out on human fibroblasts. All sol samples at five concentrations (CeO-NPs-1 at concentrations of 10^−2^, 10^−3^, 10^−4^, 10^−5^, and 10^−6^ M and CeO-NPs-2 at concentrations of 10^−2^, 10^−3^, 10^−4^, 10^−5^, and 10^−6^ M) were examined in at least 12 well plates in each of the tests (MTT, counting, and dead cell evaluation). The control point was after 72 h of cocultivation of sols with fibroblasts.

Based on the results on fibroblasts, 4 out of 5 concentrations of both nanocerium species (10^−3^, 10^−4^, 10^−5^, and 10^−6^ M) were selected due to the identified cytotoxicity of the 10^−2^ M sol concentrations and their likely unsuitability for medical dressings.

The selected sol concentrations were further investigated on MSC cell lines and human keratinocytes. The results of studies at 2 control points are presented: after 48 and 72 h of cocultivation. 

### 4.2. Preparation and Characterization of Nanocerium Sols

Nanocerium synthesis methods were chosen for their simplicity, low cost, high reproducibility, and scalability. Dissolution techniques are well established for the preparation of CeO_2_-based biomaterials.

For the synthesis of cerium sols, the following reagents were used without any additional purification: cerium (IV) ammonium nitrate (Sigma-Aldrich, St. Louis, MO, USA, SKU#215473, ACS reagent, ≥98.5% (Merck KGaA, Darmstadt, Germany)), ammonium citrate dibasic (Sigma-Aldrich, St. Louis, MO, USA, SKU 25102, puriss., ≥98% (Merck KGaA, Darmstadt, Germany)), cerium nitrate hexahydrate (>99.99%, Lankhit, Moscow, Russia), aqueous ammonia (puriss. Khimmed, Moscow, Russia), citric acid monohydrate (Sigma-Aldrich, St. Louis, MO, USA, SKU #C1909, ACS reagent, ≥99.0% (Merck KGaA, Darmstadt, Germany)), and isopropyl alcohol (≥99.5%, Khimmed, Moscow, Russia). Prior to the syntheses, the actual molar masses of cerium compounds were determined gravimetrically.

#### 4.2.1. Preparation of Aqueous Cerium Dioxide Sols by Citrate Method Using Cerium Nitrate

For the synthesis of CeO-NPs-1, citric acid was dissolved in 25 mL of 0.25 M cerium (III) nitrate solution to obtain a mixed solution with a Ce(NO_3_)_3_:citric acid molar ratio of 1:1. The resulting solution was rapidly added to 100 mL of 3 M aqueous ammonia solution and continuously stirred. The final mixture was stirred for 12 h. An excess of isopropanol was added to the obtained sol, yielding a white precipitate. The precipitate was further washed with isopropanol several times and dried in air at 60 °C. Cerium sol was prepared by dispersing the dry powder in distilled water [59,62]. 

CeO_2_ sols with concentrations of 10^−2^, 10^−3^, 10^−4^, 10^−5^, and 10^−6^ mol/L were prepared in measuring tubes by diluting the colloidal solution to a concentration of 0.38 mol/L with distilled water. The pH of the prepared sols was 6.5.

Since the present work is a step towards the development and commercialization of a medical device for wound healing, we considered it important to follow biological pH values. It is known that the pH of the environment of chronic long-term non-healing wounds is in the range of 7.4–8.9, and since regeneration rates are significantly reduced in an alkaline environment, we chose a target pH of 7 for the synthesized sols. To stabilize and buffer the sols, citric acid was used in molar excess (1:2), at which time the sol began to opalesce. Then, 3 M ammonia solution was added dropwise to the colloidal solution of CeO_2−x_-nH_2_O to bring it to the specified pH value of 7.

#### 4.2.2. Preparation of Aqueous Cerium Dioxide Sols by Thermal Hydrolysis Method Using Ammonium Ceric Nitrate

The second method for the synthesis of cerium sol (CeO-NPs-2) was based on the hydrothermal hydrolysis of ammonium ceric nitrate at 95 °C. For the synthesis, 40 mL of a 0.18 M aqueous solution of (NH_4_)_2_Ce(NO_3_)_6_ (CAN) was placed in a 100 mL polytetrafluoroethylene autoclave and subjected to hydrothermal treatment at 95 °C for 24 h. After the treatment, the autoclave was cooled in air, and the solid phase was separated by centrifugation for 10 min at 20,000 rpm, washed three times with isopropanol, and redispersed in distilled water. The resulting CeO_2_ sol was boiled for 2 h to remove traces of isopropanol [60]. The sol was stabilized by adding citric acid, with a CeO_2_:citric acid molar ratio if 1:2. The pH of the CeO-NPs-2 sol was further adjusted to pH 7 with diluted aqueous ammonia.

Both synthesis protocols involved washing the nanoparticles to remove the reactants. 

By diluting the concentrated solution with distilled water, samples of CeO-NPs-2 sols were prepared at concentrations of 10^−2^, 10^−3^, 10^−4^, 10^−5^, and 10^−6^ mol/L.

#### 4.2.3. The Characteristic of CeO_2_ Nanoparticles

**Figure 11:**
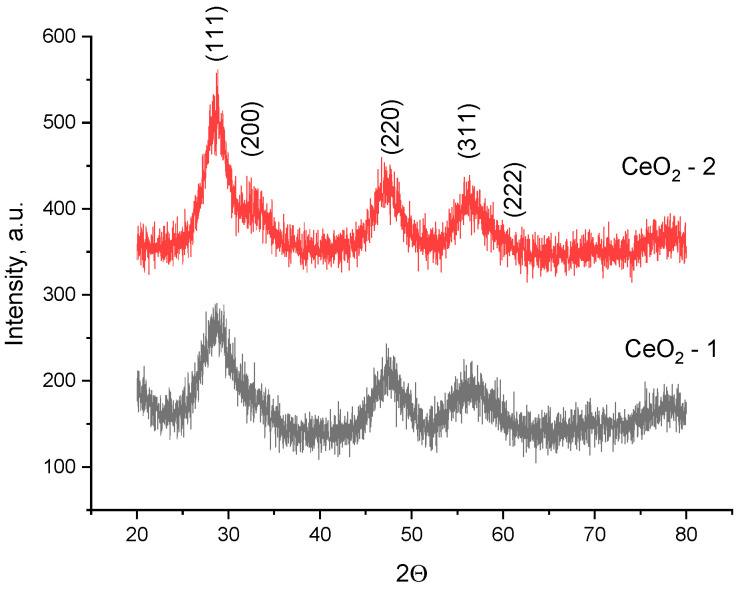
X-ray diffraction analysis patterns of CeO-NPs-1 (gray) and CeO-NPs-2 (red).

The results of an X-ray diffraction analysis of the synthesized nanoparticles confirmed the ultra-small size and crystalline nature of the CeO_2_ nanoparticles (Figure 11). The X-ray diffraction pattern is characteristic of the cubic $Fm\bar{3}m$ structure of cerium oxide (PDF2 #34–394). 

**Figure 12:**
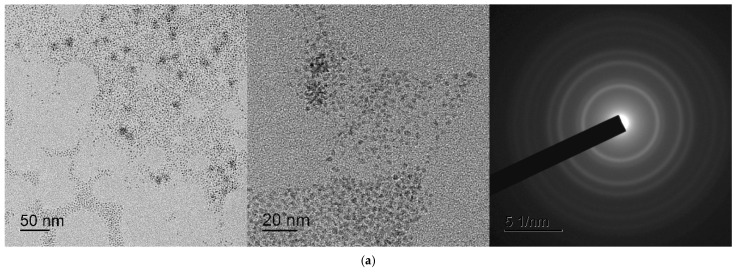

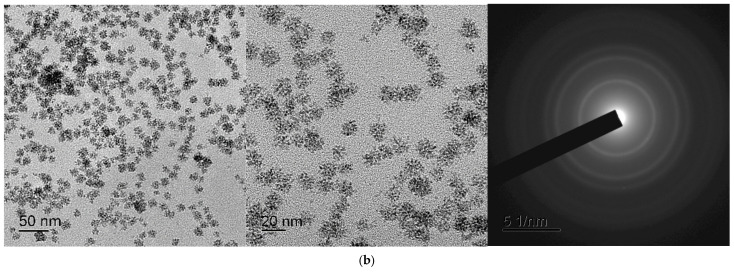
TEM images of cerium nanoparticles: (**a**) CeO_2_ precipitate redispersed in water (CeO-NPs-1); (**b**) CeO_2_ precipitate redispersed in water (CeO-NPs-2).

The size and shape of CeO_2_ nanoparticles were determined by transmission electron microscopy (TEM) using a Leo912 AB Omega (Carl Zeiss, Oberkochen, Germany) electron microscope equipped with an electron energy loss spectrometer (EELS) operating at an accelerating voltage of 100 kV. According to transmission electron microscopic data (Figure 12), the CeO-NPs-1 sample (Figure 12a) consists of weakly aggregated particles with a diameter of 3–5 nm, in agreement with the powder X-ray diffraction data. The CeO-NPs-2 sample (Figure 12b) obtained by hydrolysis of ammonium hexanitratocerate(IV) under hydrothermal conditions consists of small aggregates with a diameter of 15–20 nm, consisting of particles of 2–3 nm. The electron diffraction patterns of the selected area are typical of CeO_2_ and coincide with X-ray diffraction patterns.

**Figure 13:**
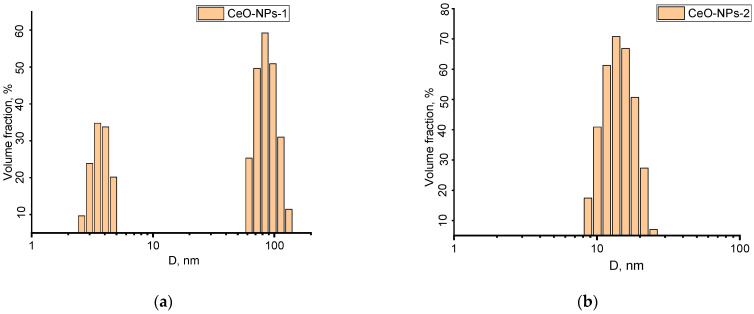
Hydrodynamic diameter of the nanoparticles in the CeO-NPs-1 (**a**) and CeO-NPs-2 (**b**) sols.

According to the dynamic light scattering data, sample 1 consisted of both individual cerium dioxide particles (3–4 nm) and their aggregates, the diameter of which ranged from 60 to 130 nm (Figure 13). For sample 2, the hydrodynamic diameter of the aggregates ranged from 8 to 20 nm.

For each cerium sol, the concentration of nanoparticles was determined gravimetrically. To this end, 4 mL of a sol sample was introduced into a dry alumina crucible, and the sol was dried in an oven and further annealed at 900 °C in air for 2 h. The weight of the formed CeO_2_ was used to determine the concentrations of the sols.

### 4.3. Methods of Evaluation of the Effect of Different Synthesis Methods and Concentrations of Cerium Oxide Nanoparticle Sols on Cytotoxicity, as Well as the Metabolic and Proliferative Activity of Human Fibroblast Cell Culture

#### 4.3.1. The Characteristics of the Fibroblast Cell Line

Experiments were performed on the BJTERT cell line, which is a modified line of primary human fibroblasts obtained by the injection of a specific genetic element, i.e., the human catalytic subunit of the telomerase enzyme (telomerase reverse transcriptase (hTERT)). These modifications were made in order to obtain cell lines convenient for experimental work because cells taken from primary material have a limited number of divisions, limiting the performance of scientific research.

hTERT-modified human fibroblasts retain all other properties of normal cells and are a laboratory analog of healthy cells. The cell source was the foreskins of newborn. 

The foreskin fibroblast cell line (immortalized hTERT) was obtained by transfection of the BJ foreskin fibroblast cell line with plasmid pGRN145 expressing hTER. 

Line origin: American collection of ATCC typed cultures (Manassas, VA, USA).

#### 4.3.2. Cell Cultivation

Cells were cultured in commercially treated Petri dishes (diam. 100 mm, Corning, New York, USA) in DMEM medium (Dulbecco’s Modified Eagle’s Medium, Paneco, Moscow, Russia) supplemented with 10% fetal calf serum (Global Kang Biotechnology, Beijing, China), 0.32 mg/mL glutamine (Paneco, Moscow, Russia), 1% penicillin, and 1% streptomycin (Paneco, Moscow, Russia).

Culture Petri dishes with cells were kept in a CO_2_ incubator (Binder, Tuttlingen, Germany) under standard controlled conditions (5% CO_2_, 37 °C). Cells were passaged every 7 days according to the standard protocol using DPBS buffer (Dulbecco’s phosphate-buffered saline, Gibco, Thermo Fisher Scientific, Waltham, MA, USA) and 0.25% trypsin–EDTA solution (Paneco, Moscow, Russia); the medium was changed every 3 days.

Control of fibroblasts for the study, as well as visualization of cells with their photofixation during the experiment, was carried out by light microscopy. A “Micromed I LUM” inverted luminescent microscope (Micromed, St.-Petersburg, Russia) with polarizing lenses and magnification of ×10 or ×20 was used for this purpose.

For experiments, BJTERT cells were seeded in 24-well plates (Thermo Fisher Scientific, Waltham, MA, USA) at a cell concentration of 5 × 10^4^ cells/mL. Cells in suspension were counted using a Countess II Automated Cell Counter and Cell Viability Analyzer (Thermo Fisher Scientific, Waltham, MA, USA). The studied substances were applied when the cells reached 70% confluency, which was achieved 24 h after cell seeding and incubation under standard controlled conditions (5% CO_2_, 37 °C). Incubation was continued for 72 h after the application of the substances.

#### 4.3.3. Methodology of the MTT Test

The MTT test is a common colorimetric method to determine metabolic activity and the number of viable cells, as well as to indirectly study the proliferative activity of the application of substances. This method is based on the tranformation of MTT reagent (3-(4,5-dimethylthiazol-2-yl)-2–5-diphenyl-tetrazolium bromide) by NADPH-H-dependent oxidoreductases into an optically active, blue-colored substance, i.e., formazan. The amount of formazan formed by this reaction is proportional to the number of viable cells in the well.

Cells were seeded into 24-well plates at the required cell concentration of 5.0 × 10^4^/mL according to the method described above. After the addition of the test substance (nanocrystalline cerium oxide in various concentrations), incubation was continued according to the design of the experiment and taking into account the characteristic features of the test substance for 72 h.

Reagents: MTT dye (thiazolyl blue tetrazolium bromide, neoFroxx, Einhausen, Germany) was used for colorimetric tests.

After the incubation time, the culture medium was removed, and 350 μL of 3% MTT reagent was added to each well and incubated for 30 min at 37 °C. Then, the MTT working solution was removed, and 300 μL of dimethyl sulfoxide (DMSO, PanReac AppliChem, Darmstadt, Germany) was added to dissolve the precipitated formazan. The solution was incubated for 5 min on an Elmi-S4 oscillating shaker (ELMI SIA, Riga, Latvia) at room temperature, after which the contents of the wells were transferred in a volume of 100 μL into a 96-well plate for measurement of optical density at a wavelength of 540 nm on a Multiscan Labsystems spectrophotometer (Labsystems Diagnostics Oy, Vantaa, Finland). The final measurement result was expressed in relative optical density (OD) units.

Each sample (each concentration) of nanocrystalline cerium oxide was tested in 12 wells. As a control, 100 µL of water for injection was injected into 12 wells.

#### 4.3.4. Determination of Proliferative Activity, Quantitative Fibroblast Counting, and Live/Dead Test

At the end of incubation, cells were detached by trypsinization, then automatically counted using a Countess II Automated Cell Counter (ThermoScientific, Waltham, MA, USA) in special disposable plastic slides (RWD, Building, China). The device counts the cells automatically when the slide is inserted into the slide port.

In addition to counting the total number of cells, the device allowed us to determine the ratio of live and dead cells. For this purpose, a reagent (dye) was added to 15 µL of cell suspension: 15 µL of 0.4% trypan blue solution (Paneco, Moscow, Russia). The solution was mixed by pipetting. The dye is able to penetrate only through the cell membrane of dead cells and stain their contents, which makes it possible to identify dead cells. Then, 25 µL of the stained cell suspension was introduced onto a slide, which was placed in a counter for automatic counting. As a result, the total concentration of cells in a unit of volume was counted, expresses as the total in number of cells (×10^4^ cells), as well as the percentage of live vs. dead cells.

### 4.4. Methods to Evaluate the Effect of Different Synthesis Methods and Concentrations of Cerium Oxide Nanoparticle Sols on Cytotoxicity, as well as on Metabolic and Proliferative Activity on Cultures of Human Mesenchymal Stem Cells and Keratinocytes

#### 4.4.1. Cell Cultures

A study was performed on cultures of human keratinocytes (HaCaT line) and human mesenchymal stem cells (MSCs) (HaCaT line) isolated from human dental pulp (cell source: Institute of Developmental Biology of the Russian Academy Sciences, Russia). 

MSCs were cultured in DMEM/F12 medium (1:1) (PanEco, Moscow, Russia) with the addition of 10% fetal calf serum Hyclone ((ThermoScientific, Waltham, MA, USA), penicillin solution (100 units/mL) (PanEco, Moscow, Russia), and streptomycin (100 μg/mL) (PanEco, Moscow, Russia). Keratinocytes were cultured in 4.5 mg/mL high-glucose DMEM (PanEco, Moscow, Russia) with the addition of 10% fetal calf serum Hyclone ((ThermoScientific, Waltham, MA, USA) and 100 units/mL penicillin/streptomycin solution (PanEco, Moscow, Russia). Cells were cultured under CO_2_ incubator (RWD, Building, China) conditions at 37 °C in a humidified atmosphere with 5% CO_2_.

Nanocerium samples were studied 4–5 days after receiving them.

#### 4.4.2. MTT Test

Cell cultures were cultured for 48 and 72 h with nanocrystalline dioxide sol series: CeO-NPs-1 and CeO-NPs-2 at concentrations of 10^−3^, 10^−4^, 10^−5^, and 10^−6^ M. 

Cells were seeded in 96-well plates (SPL, Gyeonggi-do, Korea) in complete culture medium, and 6 h after cell seeding, the culture medium was replaced with medium containing nanoparticles at different concentrations (10^−3^–10^−6^ M). Cells with medium but without nanoparticles were used as controls. At 48 and 72 h after nanocomposite application, the medium in the wells was replaced with medium containing 3-4,5-dimethylthiazol-2-yl-2,5-diphenyltetrazol (5 mg/mL) (PanEco, Moscow, Russia), then incubated for 3 h under CO_2_ incubator conditions. Subsequently, the optical density (OD) of the formed formazan was determined at a wavelength of 540 nm using an INNO-S photometer (LTek, Gyeonggi-do, Korea).

#### 4.4.3. Live/Dead Assay

The number of dead cells after incubation with nanoparticles was analyzed by staining the cell culture with a mixture of SYTO9 and propidium iodide fluorescent dyes (ThermoFisher, Waltham, MA, USA) at a concentration of 1 μg/mL. Cells were seeded into 96-well plates (SPL, Gyeonggi-do, Korea) and cultured for 48 and 72 h in complete culture medium. Then, 6 h after cell seeding, the culture medium was replaced with medium containing nanoparticles at different concentrations (10^−3^, 10^−4^, 10^−5^, and 10^−6^ M). Cells with nanoparticle-free medium were used as controls. The medium was then replaced with medium containing the dye mixture and stained for 20 min under CO_2_ incubator conditions. After staining, cells were washed three times with phosphate–salt buffer (PanEco, Moscow, Russia) before microphotography. Image analysis and microphotography were performed on a ZOE Fluorescent Cell Imager (BioRad. Hercules, CA, USA). The test result is the number of dead cells expressed as a percentage of the control.

#### 4.4.4. Quantitative Cell Culture Counting of Human MSCs and Keratinocytes Using a Cell Counter 

The cells were cultured for 48 and 72 h in the presence of nanoparticle sols of the provided samples at different concentrations. The cells were then trypsinized and detached using 0.25% trypsin/EDTA solution (PanEco, Moscow, Russia). Cell counts were performed in 5 wells of 96-well plates (SPL, Gyeonggi-do, Korea) in a volume of 500 μL using single-use slides and a specialized RWD-C100 counter (RWD, Building, China). The number of cells was counted as a percentage of the control.

### 4.5. Statistical Analysis

Statistical processing of the results of the study on cell lines was performed using the SPSS 25.0 statistical program. 

First of all, the normality of distributions of MTT test and cell counter indices for each of the samples was assessed by the Kolmogorov-Smirnov and Shapiro–Wilk tests. Checking for normality of the results proved that investigated subgroups obeyed the law of normal distribution (*p* > 0.05). 

Descriptive statistics of continuous quantitative indices are presented as mean; std. deviation; std. error, 95% confidence interval for mean (95CI), minimum, and maximum.

One-factor analysis of variance (ANOVA) was performed for comparative analysis of different subgroups of the MTT test, as well as fibroblast counts. Posterior multiple comparisons were performed using Dunnett’s test (for comparison with controls), as well as Bonferroni’s test and Duncan’s test, according to which the samples were categorized into homogeneous subsets, with the selection of the most promising ones in terms of cell stimulation. MSC and keratinocyte cell counting test results were compared only with the control using the Mann–Whitney test. Taking into account the multiple comparisons, the differences were considered statistically significant at *p* < 0.01. 

## 5. Conclusions

The method of synthesis of nanocrystalline cerium oxide and its concentration fundamentally and significantly change the biological activity of cell cultures of different types.

The best biological activity was observed when nanocerium oxide obtained by the citrate method (from aqueous solutions of cerium (III) nitrate hexahydrate and citric acid) was added to cultures of cells involved in wound regeneration. Nanoceria obtained by hydrolysis of ammonium hexanitratocerate (IV) under conditions of thermal autoclaving at 95 °C achieved worse results.

Taking into account the different biological effects on human cells depending on the concentration of the studied substance when using nanoceria as therapeutic agents, it is obvious that the choice of concentration should be guided by the phase of the wound healing process. The most preferable concentration of CeO-NPs-1 sol for the creation of medication and stimulation of all layers of cell regeneration (MSCs, fibroblasts, and keratinocytes) is 10^−3^–10^−4^ M (closer to 10^−4^ M, taking into account maximum biocompatibility and nontoxicity).

Although the synthesized cerium oxide samples have a high degree of biocompatibility, as they do not show a significant increase in the proportion of dead cells in cultures of human fibroblasts, MSCs and keratinocytes (the percentage of dead cells does not exceed 5%, which is the value in the control), the risk of cytotoxicity, which needs additional studies, is present at high concentrations of all variants of nanocerium (10^−2^ M and higher for citrate nanocerium oxide and 10^−3^ M and higher for thermohydrolyzed nanocerium oxide). 

This study showed the significant stimulating biological effects of cerium nanoparticles on cell cultures involved in skin wound regeneration. However, the reported results do not explain the mechanisms through which this stimulation is realized. It is necessary to further study the biophysical and biochemical characteristics of these nanoparticles to answer this important question.

## Figures and Tables

**Table 1:** Descriptive statistics of MTT test results when fibroblasts were cocultured with sols of nanocrystalline cerium oxides.

Group	N	Mean	Std. Deviation	Std. Error	Percentage of Cell Viability	95% Confidence Interval for Mean		Minimum	Maximum
						Lower Bound	Upper Bound		
Control	12	0.346	0.013	0.004	100.0	0.338	0.355	0.323	0.363
CeO-NPs-1 (10^−2^ M)	12	0.349	0.009	0.003	100.9	0.343	0.355	0.337	0.366
CeO-NPs-1 (10^−3^ M)	12	0.466	0.015	0.004	134.7	0.456	0.475	0.432	0.490
CeO-NPs-1 (10^−4^ M)	12	0.437	0.020	0.006	126.3	0.425	0.450	0.409	0.467
CeO-NPs-1 (10^−5^ M)	12	0.395	0.021	0.006	114.2	0.381	0.408	0.367	0.425
CeO-NPs-1 (10^−6^ M)	12	0.372	0.011	0.003	107.5	0.365	0.379	0.356	0.393
CeO-NPs-2 (10^−2^ M)	12	0.274	0.012	0.003	79.2	0.266	0.281	0.259	0.294
CeO-NPs-2 (10^−3^ M)	12	0.373	0.017	0.005	107.8	0.362	0.383	0.342	0.405
CeO-NPs-2 (10^−4^ M)	12	0.416	0.036	0.010	120.2	0.393	0.438	0.353	0.468
CeO-NPs-2 (10^−5^ M)	12	0.359	0.023	0.006	103.8	0.345	0.373	0.331	0.399
CeO-NPs-2 (10^−6^ M)	12	0.456	0.040	0.011	131.8	0.430	0.481	0.387	0.526
Total	132	0.386	0.058	0.005	111.6	0.376	0.396	0.259	0.526

**Table 2:** Descriptive statistics of fibroblast count test after 72 h of coculture with nanocrystalline cerium oxide sols (×10^4^ cells).

Group	N	Mean	Std. Deviation	Std. Error	95% Confidence Interval for Mean		Minimum	Maximum
					Lower	Upper		
Control	12	13.58	3.13	0.90	11.58	15.57	7.62	18.2
CeO-NPs-1 (10^−2^ M)	12	5.28	2.16	0.62	3.90	6.65	1.17	8.8
CeO-NPs-1 (10^−3^ M)	12	7.84	2.96	0.86	5.95	9.72	2.35	11.1
CeO-NPs-1 (10^−4^ M)	12	8.31	2.52	0.72	6.71	9.91	4.69	14.1
CeO-NPs-1 (10^−5^ M)	12	10.12	3.96	1.14	7.60	12.63	4.11	18.2
CeO-NPs-1 (10^−6^ M)	12	7.49	3.24	0.94	5.43	9.55	2.93	12.3
CeO-NPs-2 (10^−2^ M)	12	6.21	2.34	0.67	4.72	7.69	3.52	11.1
CeO-NPs-2 (10^−3^ M)	12	12.17	5.59	1.61	8.62	15.72	4.11	25.2
CeO-NPs-2 (10^−4^ M)	12	10.45	3.07	0.88	8.50	12.39	6.45	16.4
CeO-NPs-2 (10^−5^ M)	12	8.71	2.61	0.75	7.05	10.36	5.28	14.7
CeO-NPs-2 (10^−6^ M)	12	13.11	4.38	1.26	10.33	15.89	7.04	18.8
Total	132	9.39	4.20	0.37	8.66	10.11	1.17	25.2

**Table 3:** Descriptive statistics of MTT test results when MSCs were cocultured with sols of nanocrystalline cerium oxides.

Time	Group	N	Mean	Percentage of Cell Viability	Std. Deviation	Std. Error	95% Confidence Interval for Mean		Minimum	Maximum
							Lower Bound	Upper Bound		
48 h	Control	8	0.698	100.0	0.047	0.017	0.66	0.74	0.628	0.765
	CeO-NPs-1 (10^−3^ M)	8	0.673	96.4	0.045	0.016	0.63	0.71	0.580	0.718
	CeO-NPs-1 (10^−4^ M)	8	0.785	112.5	0.029	0.010	0.76	0.81	0.726	0.817
	CeO-NPs-1 (10^−5^ M)	8	0.823	117.9	0.042	0.015	0.79	0.86	0.755	0.866
	CeO-NPs-1 (10^−6^ M)	8	0.748	107.2	0.041	0.014	0.71	0.78	0.706	0.815
	CeO-NPs-2 (10^−3^ M)	8	0.687	98.4	0.038	0.014	0.65	0.72	0.621	0.746
	CeO-NPs-2 (10^−4^ M)	8	0.787	112.8	0.019	0.007	0.77	0.80	0.749	0.813
	CeO-NPs-2 (10^−5^ M)	8	0.787	112.8	0.044	0.016	0.75	0.82	0.729	0.833
	CeO-NPs-2 (10^−6^ M)	8	0.709	101.6	0.021	0.007	0.69	0.73	0.677	0.741
	Total in 48 h	72	0.744	106.6	0.062	0.007	0.73	0.76	0.580	0.866
72 h	Control	8	0.977	100.0	0.063	0.022	0.92	1.03	0.908	1.090
	CeO-NPs-1 (10^−3^ M)	8	0.992	101.5	0.040	0.014	0.96	1.03	0.922	1.053
	CeO-NPs-1 (10^−4^ M)	8	1.071	109.6	0.040	0.014	1.04	1.10	0.976	1.099
	CeO-NPs-1 (10^−5^ M)	8	1.097	112.3	0.032	0.011	1.07	1.12	1.033	1.129
	CeO-NPs-1 (10^−6^ M)	8	0.957	98.0	0.051	0.018	0.91	1.00	0.888	1.012
	CeO-NPs-2 (10^−3^ M)	8	0.821	84.0	0.100	0.035	0.74	0.90	0.696	1.039
	CeO-NPs-2 (10^−4^ M)	8	1.148	117.5	0.101	0.035	1.06	1.23	1.064	1.376
	CeO-NPs-2 (10^−5^ M)	8	1.096	112.2	0.122	0.043	0.99	1.20	0.970	1.310
	CeO-NPs-2 (10^−6^ M)	8	0.893	91.4	0.174	0.061	0.75	1.04	0.618	1.206
	Total in 72 h	72	1.006	103.0	0.133	0.016	0.97	1.04	0.618	1.376

**Table 4:** Descriptive statistics of MTT test results when keratinocytes were cocultured with sols of nanocrystalline cerium oxides.

Time	Group	Mean	Percentage of Cell Viability	Std. Deviation	Std. Error	95% Confidence Interval for Mean		Minimum	Maximum
						Lower	Upper		
48 h	Control	0.389	100.0	0.064	0.020	0.343	0.434	0.317	0.540
	CeO-NPs-1 (10^−3^ M)	0.782	201.0	0.128	0.045	0.675	0.888	0.636	0.981
	CeO-NPs-1 (10^−4^ M)	0.378	97.2	0.024	0.009	0.355	0.399	0.326	0.397
	CeO-NPs-1 (10^−5^ M)	0.391	100.5	0.025	0.009	0.367	0.413	0.354	0.433
	CeO-NPs-1 (10^−6^ M)	0.378	97.2	0.039	0.015	0.341	0.414	0.308	0.433
	CeO-NPs-2 (10^−3^ M)	0.377	96.9	0.061	0.019	0.333	0.421	0.320	0.499
	CeO-NPs-2 (10^−4^ M)	0.394	101.3	0.040	0.015	0.356	0.430	0.364	0.480
	CeO-NPs-2 (10^−5^ M)	0.379	97.4	0.025	0.009	0.355	0.401	0.344	0.411
	CeO-NPs-2 (10^−6^ M)	0.378	97.2	0.043	0.016	0.338	0.418	0.321	0.452
	Total in 48 h	0.429	110.3	0.140	0.016	0.395	0.461	0.308	0.981
72 h	Control	0.435	100.0	0.028	0.009	0.415	0.455	0.372	0.459
	CeO-NPs-1 (10^−3^ M)	1.479	340.0	0.404	0.122	1.207	1.751	0.833	2.134
	CeO-NPs-1 (10^−4^ M)	1.261	289.9	0.263	0.088	1.058	1.463	0.951	1.736
	CeO-NPs-1 (10^−5^ M)	0.616	141.6	0.103	0.039	0.520	0.710	0.419	0.730
	CeO-NPs-1 (10^−6^ M)	0.677	155.6	0.095	0.036	0.588	0.764	0.530	0.775
	CeO-NPs-2 (10^−3^ M)	0.307	70.6	0.085	0.030	0.235	0.378	0.206	0.395
	CeO-NPs-2 (10^−4^ M)	0.462	106.2	0.063	0.020	0.417	0.507	0.382	0.569
	CeO-NPs-2 (10^−5^ M)	0.551	126.7	0.109	0.034	0.473	0.628	0.399	0.679
	CeO-NPs-2 (10^−6^ M)	0.533	122.5	0.107	0.040	0.434	0.632	0.390	0.688
	Total in 72 h	0.726	166.9	0.439	0.049	0.627	0.824	0.206	2.134

## Data Availability

The database and the indicators analyzed in the work are held by the first author Ekaterina Silina (silinaekaterina@mail.ru).
