# Correction: Silina et al. Influence of the Synthesis Scheme of Nanocrystalline Cerium Oxide and Its Concentration on the Biological Activity of Cells Providing Wound Regeneration. *Int. J. Mol. Sci.* 2023, *24*, 14501

**DOI:** 10.3390/ijms27041810

**Published:** 2026-02-13

**Authors:** Ekaterina V. Silina, Victor A. Stupin, Natalia E. Manturova, Olga S. Ivanova, Anton L. Popov, Elena A. Mysina, Elena B. Artyushkova, Alexey A. Kryukov, Svetlana A. Dodonova, Maria P. Kruglova, Alexey A. Tinkov, Anatoly V. Skalny, Vladimir K. Ivanov

**Affiliations:** 1 Institute of Biodesign and Modeling of Complex Systems, Center of Bioelementology and Human Ecology, I.M. Sechenov First Moscow State Medical University (Sechenov University), 119991 Moscow, Russia; marykruglova@live.ru (M.P.K.); tinkov.a.a@gmail.com (A.A.T.); skalny3@gmail.com (A.V.S.); 2 Department of Hospital Surgery, Pirogov Russian National Research Medical University, 117997 Moscow, Russia; stvictor@bk.ru; 3 Department of Plastic and Reconstructive Surgery, Cosmetology and Cell Technologies, Pirogov Russian National Research Medical University, 117997 Moscow, Russia; manturovanatali@yandex.ru; 4 Frumkin Institute of Physical Chemistry and Electrochemistry, Russian Academy of Sciences, 119071 Moscow, Russia; runetta05@mail.ru; 5 Institute of Theoretical and Experimental Biophysics, Russian Academy of Sciences, 142290 Pushchino, Russia; antonpopovleonid@gmail.com (A.L.P.); mironova_e27@rambler.ru (E.A.M.); 6 Research Institute of Experimental Medicine, Kursk State Medical University, 305041 Kursk, Russia; eartyushkova@mail.ru (E.B.A.); krukovaa@kursksmu.net (A.A.K.); dodonovasa@kursksmu.net (S.A.D.); 7 Laboratory of Ecobiomonitoring and Quality Control, Yaroslavl State University, 150003 Yaroslavl, Russia; 8 Kurnakov Institute of General and Inorganic Chemistry, Russian Academy of Sciences, 119991 Moscow, Russia; van@igic.ras.ru

In the original publication [1], there were some mistakes in Figures 10 and 12 as published. The corrected version of the figures appears below.

Two co-authors performed the study on HaCaT keratinocytes, and they provided identical photos of dead cells, which may be due to a technical but unprincipled error (red dead cells were almost nowhere to be found and were not visually distinguishable). This technical error showed the authors’ synthesized nanoceria worse than they are in reality. Nevertheless, the conclusion about nontoxicity (biocompatibility) is correct. The corrected Figure 10 appears below.

**Figure 10:**
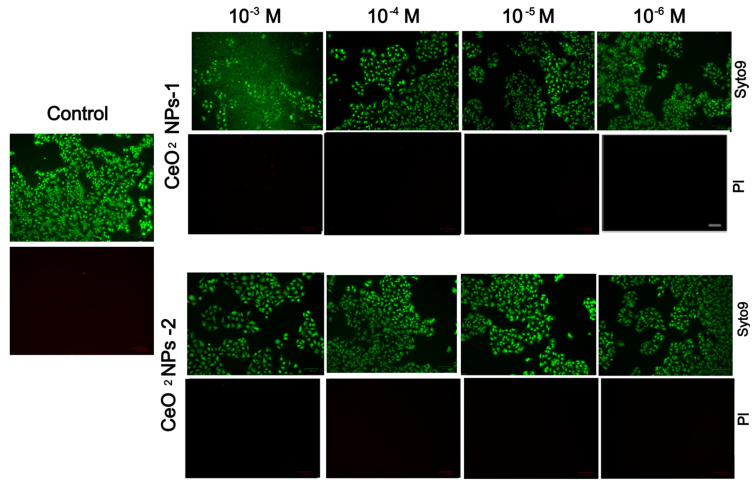
Staining of keratinocytes with SYTO9 and propidium iodide fluorescent dyes after cultivation with nanoparticles for 72 h. Quantitative analysis of the number of dead cells in relation to the total number of cells. Scale bar—100 µm.

The original electron diffraction patterns are of poor quality. The same nanocerium samples were remade using another better transmission electron microscope (JEOL JEM 2100, Akishima, Japan). Repeated studies of these samples confirmed that the location of the diffraction maxima on the ring electronograms of the samples corresponds to the CeO_2_ phase, but the quality of the electronograms was significantly improved. The corrected Figure 12 appears below.

**Figure 12:**
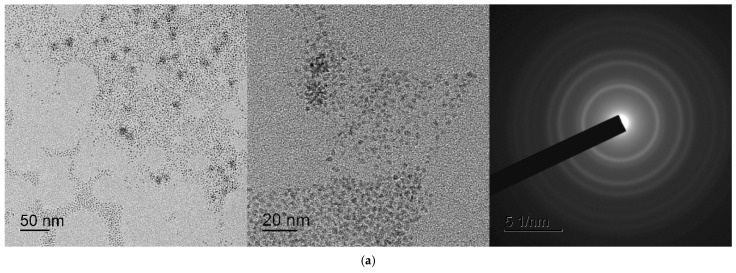

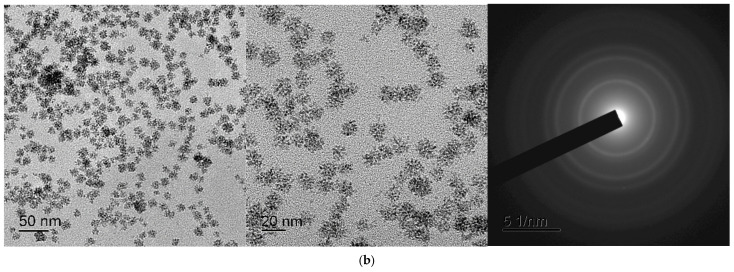
TEM images of cerium nanoparticles: (**a**) CeO_2_ precipitate redispersed in water (CeO-NPs-1); (**b**) CeO_2_ precipitate redispersed in water (CeO-NPs-2).

The authors state that the scientific conclusions are unaffected. This correction was approved by the Academic Editor. The original publication has also been updated.
